# Soluble CD73 as biomarker in patients with metastatic melanoma patients treated with nivolumab

**DOI:** 10.1186/s12967-017-1348-8

**Published:** 2017-12-04

**Authors:** Silvana Morello, Mariaelena Capone, Claudia Sorrentino, Diana Giannarelli, Gabriele Madonna, Domenico Mallardo, Antonio M. Grimaldi, Aldo Pinto, Paolo Antonio Ascierto

**Affiliations:** 1 Department of Pharmacy, University of Salerno, Via Giovanni Paolo II 132, 84084 Fisciano, SA Italy; 2 Melanoma, Cancer Immunotherapy and Innovative Therapies O.U, National Cancer Institute “G. Pascale”, Naples, Italy; 3 PhD Program in Drug Discovery and Development, University of Salerno, Fisciano, SA Italy; 4 Regina Elena National Cancer Institute, Rome, Italy

**Keywords:** Soluble CD73, Melanoma, Nivolumab, Immunotherapy, Biomarker

## Abstract

**Background:**

Nivolumab is an anti-PD1 checkpoint inhibitor active in patients with advanced melanoma and as adjuvant therapy in high-risk metastatic melanoma patients.

**Methods:**

In this single-center retrospective analysis, we investigated the CD73 enzyme activity in patients with metastatic melanoma stage IV and its correlation with the response to nivolumab. The soluble CD73 (sCD73) enzyme activity was measured in the serum of 37 melanoma patients before receiving nivolumab and the Harrel’s C index was used to find the best cut-off for this biomarker. The multivariate Cox proportional hazard model was used to evaluate the prognostic value of CD73 enzyme activity for survival and progression-free survival.

**Results:**

Our results show that high levels of sCD73 enzyme activity were significantly associated with poor overall survival and progression-free survival in patients with metastatic melanoma. The median progression–free survival was 2.6 months [95% confidence interval (CI) 1.9–3.3] in patients with high sCD73 enzyme activity (> 27.8 pmol/min/mg protein), and 14.2 months (95% CI 4.6–23.8) in patients with lower CD73 enzyme activity, when patients were follow-up for a median of 24 months range. The median overall survival was not reached in patients with low sCD73 activity (< 27.8 pmol/min/mg protein) compared with 6.1 months (95% CI 0–14.8) in patients with higher sCD73 activity. In multivariate analyses, the sCD73 enzyme activity emerged as the strongest prognostic factor for overall survival and progression-free survival. Elevated basal levels of sCD73 enzyme activity, before starting nivolumab treatment, were associated with lower response rates to therapy.

**Conclusions:**

We observed a significant association between the activity of sCD73 in the blood and clinical outcomes in patients with metastatic melanoma stage IV, receiving nivolumab. Although our results need to be confirmed and validated, we suggest that sCD73 might be used as serologic prognostic biomarker. Potentially evaluating sCD73 enzyme activity in the peripheral blood before treatment could help to estimate the response to nivolumab.

## Background

Over the past years’ treatment of metastatic melanoma has been revolutionized with immunotherapy, including inhibitors of modulatory receptors on T cells (also known as “immune checkpoints”), that are able to significantly improve T-cell mediated immune response against cancer cells [1]. The first class of immune checkpoints approved by US Food and Drug Administration (FDA) for metastatic melanoma patients included an antibody anti-Cytotoxic T Lymphocyte Antigen-4 (CTLA-4) (ipilimumab), a co-inhibitory receptor expressed on the surface of T-cells. Ipilimumab is able to enhance T-cell proliferation and activation [2] and limit Tregs activity [3] inducing long-term regression of melanoma, although the therapeutic benefit of ipilimumab is limited to a small subset of patients [4–6]. Severe immune-related adverse events are common in patients receiving ipilimumab, and occasionally toxicity may cause severe morbidity or even mortality [7–10].

Programmed cell death protein-1 (PD-1) and its ligand PD-L1 are novel therapeutic immune checkpoint targets for cancer therapy [11]. PD-1 is a receptor expressed on CD4+T cells and CD8+ T cells that binds its ligands PD-L1 expressed on different cell types including tumor cells, or PD-L2 on macrophages and dendritic cells, inhibiting the function of T cells [12]. Nivolumab is an antibody anti-PD1 approved for the treatment of advanced melanoma patients for monotherapy and combination therapy [13–19]. Treatment of melanoma patients with nivolumab alone or in combination with ipilimumab has proved to induce an objective response rate (44–58%) higher than ipilimumab alone and a significantly longer progression-free survival [15, 19, 20]. Grade 3 or 4 adverse events to nivolumab occur in 14.4% of patients, significantly lower than those observed in patients receiving ipilimumab (45.9%); while 17.5% of patients experience any grade treatment-related effects [21]. Similar results were observed in patients with metastatic melanoma treated with pembrolizumab, a highly selective inhibitor of PD1, also approved for metastatic melanoma patients who progressed after ipilimumab or BRAF inhibitors treatment if appropriated [22, 23].

PD-L1 expression on melanoma cells, that was supposed to be required for response to nivolumab, appears to be associated to a more favourable response to PD1/PD-L1 blocking antibodies [13, 24, 25]. However, the value of PD-L1 in melanoma as prognostic biomarker remains controversial because other studies have demonstrated that patients with PD-L1 negative tumours can also respond to anti-PD-1 pathway therapy [17, 26, 27]. Thus, the identification of biomarkers that correlates with rates of response to PD-1 blockade is a challenge for oncologists to define the subsets of patients who will likely benefit from this therapy [28, 29].

In this single-center retrospective study, we evaluated the association of sCD73 enzyme activity with clinical outcomes of patients with metastatic melanoma, receiving nivolumab. CD73, or ecto-5’-nucleotidase, is an enzyme which hydrolyses the extracellular AMP into adenosine, a potent anti-inflammatory mediator that critically impairs the anti-tumor immune response [30, 31]. CD73 is up-regulated in different types of human solid tumors (reviewed in Ref [32]). The over-expression of tumor CD73 is in general associated with worse overall survival or progression-free survival, as recently showed in a meta-analysis and systematic review conducted by Wang and collaborators [33]. CD73 is expressed on the surface of many cell types, including immune cells, endothelial cells, and tumor cells. Interestingly, recent data indicate that a soluble form of CD73 (sCD73) in the plasma also exists, and its levels are significantly increased in the plasma of cancer patients compared with healthy individuals [34, 35], as well as in patients with acute inflammatory pancreatitis [36].

Here, we observed that melanoma patients stage IV with elevated basal serum level of sCD73 enzyme activity, before starting nivolumab treatment, had a lower response rate to nivolumab, shorter survival and higher rates of progression of disease. Patients obtaining partial response (PR) to nivolumab or stable disease had low levels of sCD73 activity in the serum, thus suggesting a predictive role for sCD73. Although it is far from a validated predictive biomarker, plasma sCD73 enzyme activity could play a role in defining the subsets of patients who will benefit from a therapy with nivolumab.

## Methods

### Patients characteristics

This retrospective single-center study included a total of 37 patients (male, n = 21; female n = 16) aged > 18 years with metastatic stage IV melanoma and samples were collected between January 1st 2015 and December 31st 2016 at the Department of Melanoma, Cancer Immunotherapy and Innovative Therapies Unit. N = 13 patients of 37 patients (35%) had BRAF V600-mutant melanoma, n = 19 patients (51%) were wild-type for BRAF, while n = 5 patients included in this study had unknown BRAF status. 27/37 patients (73%) did not present brain metastases, while n = 7 patients (19%) had brain metastases. N = 24 patients (65%) had M1c disease and n = 19 (51%) had an elevated lactate dehydrogenase level (LDH > 480 International Units [IU]/L). Nivolumab at the dosage of 3 mg/kg was administered in patients who had previously received ipilimumab alone or in combination with a BRAF inhibitor (n = 7 patients), every 2 weeks until disease progression or unacceptable toxicity appeared. Tumor response was evaluated at 12 weeks and then every 12 weeks until progression or the discontinuation of treatment according to the Response Evaluation Criteria In Solid Tumors (RECIST), version1.1. The overall response rate (ORR) to nivolumab was 24% (9/37: 0 patients with complete response [CR] and 9 patients with partial response). The disease control rate (DCR) to nivolumab was 46% (17/37: 0 patients CR, 9 PR and 8 with stable disease [SD]). 46% of patients (17/37) showed progressive disease (PD); while 3 patients died before the assessment of response to treatment.

### CD73 enzyme activity

The CD73 enzyme activity of hydrolysing AMP to adenosine and inorganic phosphate was determined in sera by measuring the concentrations of inorganic phosphate (P*i*) with the Malachite Green assay kit (CliniSciences, Italy), as previously described [37–39]. Serum was obtained from blood sample of each patients withdrawn before starting nivolumab treatment. Briefly, serum samples (100 µg of proteins) were incubated in assay medium containing MgCl_2_ (10 Mm), NaCl (120 mM), KCl (5 mM), glucose (60 mM), Tris-HCl (50 mM), pH 7.4 for 10 min. AMP (2 mM) was added as substrate and samples kept at 37°C for 40 min. To stop the reaction trichloroacetic acid (TCA, final concentration 5% w/v) was added. The concentration of inorganic phosphate (P*i*) released during the hydrolysis of AMP was measured using the Malachite Green assay according to the manufacturer’s instructions. To have the net value of P*i* produced following enzymatic reaction, aspecific P*i* released in absence of AMP in each sample was subtracted from the value obtained following incubation with AMP. To determine specificity, experiments were also performed in the presence of the CD73 inhibitor, adenosine 5’-(a,b-methylene) diphosphate (APCP; Tocris Bioscience, Bristol, U.K.) (40–100 µM), which is a non-hydrolysable ADP analogue. For these experiments, samples were incubated with APCP in assay medium for 30 min at 37°C before adding AMP. All samples were run in triplicate; results were expressed as P*i* released (pmol/min/mg protein). All reagents, as not indicated otherwise, were from Sigma-Aldrich S.r.l. (Milan, Italy).

### Statistical analysis

Differences in sCD73 enzyme activity values across patients’ characteristics were evaluated with the Mann–Whitney non parametric test. Chi square test was used to study associations among categorical variables. Survival curves were estimated with the Kaplan–Meier method and differences evaluated with the log-rank test. Associations between CD73 values and survival times were measured with the Harrel’c index. Best cut-off values were located with an R routine implemented on the online software (Cut-off Finder) which maximize differences in survival between the two groups. Hazard Ratios (HR) and their 95% confidence intervals (95% CI) were calculated using the proportional hazard model. Multivariate analysis was performed to identify independent factors associated with survival times.

## Results

### sCD73 enzymatic activity in patients with metastatic melanoma

In this study we retrospectively analysed the basal levels of soluble CD73 (sCD73) enzyme activity in the serum samples of patients with metastatic melanoma, before starting nivolumab treatment. The median enzyme activity of sCD73 was analysed in relationship to gender, age, BRAF status (wild-type versus mutated), brain metastases, LDH levels and number of previous therapy line, as summarized in Table 1. 70% of the patients presented detectable CD73 enzyme activity; whilst some samples presented no detectable CD73 enzyme activity. Elevated sCD73 activity was associated with male gender (P = 0.007), but not with other variables (Table 1). Indeed, patients with BRAF V600-mutant melanoma had values of sCD73 enzyme activity similar to those measured in BRAF wild-type melanoma (P = 0.99) (Table 1). No significant differences in the activity of sCD73 was found between patients with or without brain metastases (P = 0.84), in patients with normal LDH versus patients with elevated LDH values (P = 0.11), or in relationship to the number of previous line of treatment (P = 0.28) (Table 1).

**Table 1:** sCD73 activity in metastatic melanoma patients

	n	SCD73 activity pmol/min/mg protein [median (range)]	P value^a^
Gender			
Male	21	26.7 (0.0–255.3)	0.007
Female	16	0.0 (0.0–75.8)	
Age (years)			
< 62	18	21.9 (0.0–93.7)	0.16
≥ 62	19	6.4 (0.0–255.3)	
BRAF status			
Wt	19	16.5 (0.0–90.9)	0.99
Mutated	13	13.9 (0.0–255.3)	
Brain mets			
Yes	7	16.0 (0.0–90.9)	0.84
No	27	13.9 (0.0–255.3)	
LDH (IU/L)			
< 480	17	7.2 (0.0–90.9)	0.11
> 480	19	25.2 (0.0–255.3)	
N of previous therapy line			
2	24	10.0 (0.0–93.7)	0.28
> 2	12	19.5 (0.0–255.3)	

*WT* wild-type, *mets*: metastasis, *LDH* lactate dehydrogenase, *IU/L* International Units per Liter, *N* number

^a^Mann–Whitney U test

**Fig. 1:**
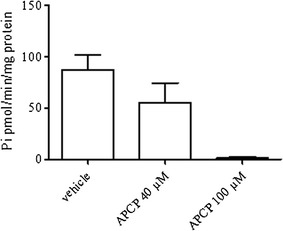
The activity of CD73 in hydrolysing AMP was inhibited in presence of the CD73 inhibitor APCP (40–100 µM). APCP was added to the samples before adding the substrate AMP. Data are mean ± S.E.M

To determine the specificity of the reaction, samples with the highest sCD73 enzyme activity were incubated with the non-hydrolysable inhibitor of CD73, APCP (40–100 µM) before adding the substrate AMP. APCP significantly reduced the accumulation of P*i* in a concentration-dependent manner [from 87.337 ± 27.995 to 55.00 ± 38.658 pmol/min/mg protein (APCP, 40 µM) and to 1.375 ± 2.428 pmol/min/mg protein (APCP, 100 µM); n = 4, P < 0.05 Mann–Whitney U test] (Fig. 1).

### sCD73 enzymatic activity and survival

In order to investigate the association between sCD73 enzymatic activity values and overall survival and progression-free survival Harrel’s C index was calculated. These values showed a good association c = 0.68 (0.45–0.92) for overall survival and c = 0.65 (0.44–0.85) for progression-free survival.

**Fig. 2:**
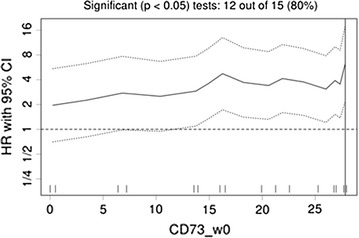
Hazard ratio (HR) and 95% confidence intervals (CI) according to different values of basal CD73 enzyme activity

To establish the sensitivity and specificity of sCD73 enzyme activity thresholds for survival the optimal cut-off value was calculated. The optimal cut-off of sCD73 activity for both overall survival and progression-free survival was 27.82 pmol/min/mg protein, as determined by Cut-off Finder online software (Fig. 2). Among patients with metastatic melanoma analysed in this study, 27/37 (73%) patients showed a cut-off value of sCD73 < 27.8 pmol/min/mg protein and 10/37 (27%) patients with sCD73 > 27.8 pmol/min/mg protein.

**Fig. 3:**
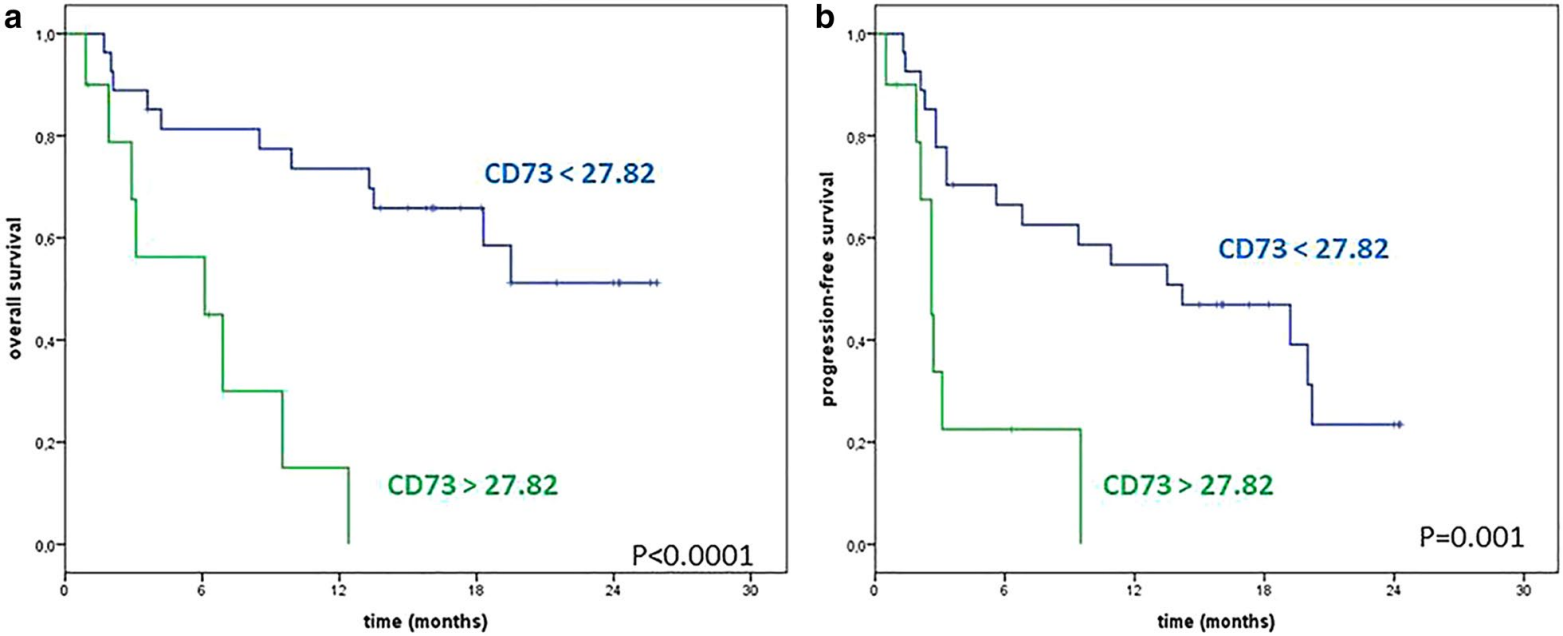
Kaplan–Meier 24-months curves of overall survival (OS; **a**) and progression-free survival (PFS; **b**) of metastatic melanoma patients treated with nivolumab, according to basal sCD73 enzyme activity. Blue line: CD73 <27.8 pmol/min/mg protein; green line: CD73 >27.8 pmol/min/mg protein

Median overall survival was not reached in patients with low values of sCD73 enzyme activity (<27.82 pmol/min/mg protein) compared with 6.1 (95% CI 0–14.8) months for patients with elevated sCD73 enzyme activity (> 27.82 pmol/min/mg protein) (Fig. 3a; P < 0.0001), when patients were follow-up for a median of 24 months. Median progression-free survival was 14.2 months (95% CI 4.6–23.8) in patients with low sCD73 enzyme activity (< 27.82 pmol/min/mg protein) and 2.6 months (95% CI 1.9–3.3) in patients with sCD73 enzyme activity higher than 27.82 pmol/min/mg protein (Fig. 3b; P = 0.001).

### sCD73 enzyme activity as possible prognostic factor in metastatic melanoma

Serum lactate dehydrogenase (LDH) is one of the most important recognized independent prognostic factor in melanoma patients with stage IV disease [40]. In line with this, univariate logistic regression was performed to evaluate the impact of gender, age, BRAF status, LDH levels, line of treatment, brain metastasis or basal activity of sCD73 on response to nivolumab. Elevated LDH levels were found to be significantly associated with worse overall survival and progression-free survival among the patients of this study (Tables 2, 3, respectively). The number of previous treatment also impacts on progression-free survival (Table 3). Interestingly, we observed that patients with high basal sCD73 enzyme activity (> 27.8 pmol/min/mg protein) had six- and four-fold increased risk of death and progression, respectively, when compared with patients with sCD73 enzyme activity < 27.8 pmol/min/mg protein (HR = 6.27; 95% CI 2.17–18.11; P = 0.001 and HR = 4.24; 95% CI 1.64–10.93; P = 0.003, respectively) (Tables 2, 3, respectively).

**Table 2:** Analyses of the associations of sCD73 activity and overall survival

	Univariate	Multivariate
	HR (95% CI) P value	HR (95% CI) P value
Gender (M vs. F)	2.08 (0.80–5.38) P = 0.13	n.s.
Age, years (≥ 62 vs. < 62)	1.09 (0.45–2.65) P = 0.85	n.s.
BRAF status (mut vs. wt)	0.53 (0.19–1.48) P = 0.22	n.s.
LDH, IU/L (≥ 480 vs. < 480)	2.72 (1.07–6.88) P = 0.03	n.s.
Line of treatment (> 2 vs. 2)	2.38 (0.96–5.94) P = 0.06	n.s.
Brain METS (yes vs. no)	1.04 (0.35–3.12) P = 0.94	n.s.
Basal sCD73 activity (≥ 27.8 vs. < 27.8)	6.27 (2.17–18.11) P = 0.001	6.27 (2.17–18.11) P = 0.001

*HR* hazard ratio, *CI* confidence interval, *n.s.* not significant, *M* male, *F* female, *MUT* mutant, *WT* wild-type, *LDH* lactate dehydrogenase, *IU/L* International Units per Liter, *METS* metastasis, *sCD73* soluble CD73

P values are from Cox proportional hazard Regression models

**Table 3:** Analyses of the associations of sCD73 activity and progression-free survival

	Univariate	Multivariate
	HR (95% CI) P value	HR (95% CI) P value
Gender (M vs. F)	1.64 (0.73–3.70) P = 0.23	n.s.
Age, years (≥ 62 vs. < 62)	1.30 (0.59–2.89) P = 0.52	n.s.
BRAF status (mut vs. wt)	0.72 (0.30–1.69) P = 0.45	n.s.
LDH, IU/L (≥ 480 vs. < 480)	2.37 (1.04–5.39) P = 0.04	n.s.
Line of treatment (> 2 vs. 2)	2.34 (1.02–5.34) P = 0.04	n.s.
Brain METS (yes vs. no)	1.33 (0.53–3.36) P = 0.54	n.s.
Basal sCD73 activity (≥ 27.8 vs. < 27.8)	4.24 (1.64–10.93) P = 0.003	4.24 (1.64–10.93) P = 0.003

*HR* hazard ratio, *CI* confidence interval, *n.s.* not significant, *M* male, *F* female, *MUT* mutant, *WT* wild-type, *LDH* lactate dehydrogenase, *IU/L* International Units per Liter, *METS* metastasis, *sCD73* soluble CD73

P values are from Cox proportional hazard Regression models

At multivariate analysis, sCD73 enzyme activity remained the only factor associated with survival, both overall and progression-free (HR = 6.27; 95% CI 2.17–18.11; P = 0.001) (Tables 2, 3, respectively).

### The sCD73 enzyme activity correlates with clinical response to nivolumab in metastatic melanoma patients

We next found that there is also an association between the response of patients to nivolumab and the value of the enzyme activity of sCD73 measured in the serum before treatment with nivolumab, compared with patients who do not respond to treatment. All patients considered in this study who showed a PR to nivolumab or stable disease (SD) had low levels of sCD73 enzyme activity before starting the treatment (Table 4). Accordingly, the DCR to nivolumab, defined as the proportion of patients with CR, PR and SD, was significantly associated with low pre-treatment sCD73 enzyme activity (< 27.8 pmol/min/mg protein) (P = 0.001) (Table 4). Conversely, almost half of the patients who progressed (PD) after nivolumab treatment had elevated basal sCD73 enzyme activity (Table 4).

**Table 4:** Response to treatment related to sCD73 enzyme activity levels

	sCD73 < 27.8 pmol/min/mg protein	sCD73 > 27.8 pmol/min/mg protein	Pts
CR	–	–	0
PR	9 (100%)	0	9
SD	8 (100%)	0	8
PD	9 (52.9%)	8 (47.1%)	17
DCR (CR + PR + SD)	17 (100%)	0	17

*Pts* patients, *CR* complete response, *PR* partial response, *SD* stable disease, *PD* progressive disease, *DCR* disease control rate

These results suggest that elevated serum levels of sCD73 enzyme activity could be a negative predictor of response to nivolumab therapy.

## Discussion

Nivolumab and other monoclonal antibodies against PD1/PD1L pathway represent the most effective therapeutics in advanced melanoma to date. The identification of biomarkers that predict the clinical response to anti-PD1 drugs still remains a challenge for selecting patients for therapy.

This study provides preliminary evidence of a significant association between the activity of sCD73 and clinical outcomes in patients with metastatic melanoma stage IV, including overall survival and progression-free survival. Patients with elevated serum sCD73 enzyme activity had a significantly increased risk of death and disease progression compared to patients with low CD73 enzyme activity. Interestingly, we observed that the basal activity of sCD73 in the serum, determined before starting nivolumab therapy, is elevated in patients who do not respond to nivolumab treatment, while patients who benefit from nivolumab treatment had lower levels of sCD73 enzyme activity.

High expression of CD73 has been found in melanoma cell lines, associated with an invasive phenotype [41, 42] and metastasis-promoting antigens [43]. In malignant melanoma expression of CD73 is epigenetically regulated and methylation in the *NT5E* (CD73) CpG island is associated with high risk of metastasis to brain and visceral sites [44]. Very recently, although elevated tumor levels of CD73 have been found in melanoma patients with late-stage disease [45], the expression of CD73 within tumor microenvironment is heterogeneous in primary melanomas and cutaneous melanoma metastases [42], raising the question whether immunohistochemical analysis of CD73 may be a valuable prognostic factor in melanoma. Here, detectable sCD73 enzyme activity was found in the peripheral blood of 70% of the patients with stage IV melanoma and elevated levels of CD73 enzyme activity were associated with worse overall survival and progression-free survival. In the population of patients reported in this study the levels of sCD73 enzyme activity were not associated with the status of BRAF (mutated versus wild-type), the presence or not of brain metastasis, or the levels of LDH. In addition, we did not observe any significant difference in the basal sCD73 enzyme activity levels between patients previously treated with ipilimumab and those treated with BRAF inhibitors if indicated. To the best of our knowledge, this is the first observation that the activity of sCD73 determined in the peripheral blood of melanoma patients may have a value as prognostic factor, and although these results need to be confirmed and validated in larger randomized studies, they would suggest that prospectively CD73 could be used as serologic biomarker, in addition to known clinical prognostic parameters on multivariate analysis in patients with advanced melanoma. A validation in external populations, that is in progress, may also help to establish the best definitive cut-off reference for sCD73 activity-positive status and its specificity for metastatic melanoma. Nonetheless, elevated levels of sCD73 activity and expression have been found also in the serum of patients with other types of tumors [34, 35]. Perhaps, the increased levels of sCD73 in the serum might have a prognostic value extending beyond melanoma to other cancers, and it could complement the prognostic value of other biomarkers or histopathologic features to guide also the selection of patients for therapy.

Increased levels of sCD73 in the serum may probably reflect an elevated expression of CD73 within tumor microenvironment in cancer patients, most likely as consequence of tissue-associated inflammation/hypoxia [46] and possibly it may be useful to direct patients for treatment with CD73/adenosine pathway-targeted therapies. However, whether CD73 in the peripheral blood correlates with its expression in the tumor microenvironment or even with the degree of T cells infiltration and/or PD-L1 expression remains unknown.

Changes in tumor CD73 expression has been recently seen in BRAF-mutant melanoma patients receiving BRAF/MEK inhibitors and in BRAF-mutant melanoma cell lines [42, 45, 47]. Almost half of the patients treated with BRAF/MEK inhibitors showed decreased tumor CD73 expression [42, 45]. Conversely, an up-regulation on-treatment of tumor CD73 expression has been found in a subset of melanoma patients who, in most cases, had progressed after pembrolizumab therapy or MART-1 adoptive T cell transfer therapy, suggesting a possible mechanism of drug acquired resistance [42]. It is important therefore to establish in future studies whether there is any correlation between the enzymatic activity of sCD73 in the peripheral blood of patients with advanced melanoma and clinical outcomes following PD-1 blockade regardless the treatment; or whether sCD73 enzyme activity in the serum could change after receiving PD1 blocking antibodies and if this value could be still associated with response rate (regardless the changes of CD73). Studies to solve this issue are in progress.

Numerous pre-clinical studies have demonstrated that adenosinergic pathway-targeted therapies can significantly reduce tumor growth and metastasis, improving the T-cell-mediated anti-tumor response [48–52] and synergize with antibodies anti-PD1 and anti-CTLA4 [53–56], establishing the bases for ongoing clinical trials [NCT02503774 and NCT02655822]. In this study, we found that the activity of sCD73 determined in the serum before treatment with nivolumab is inversely associated with objective response to nivolumab, leading us to suggest this marker as possible candidate to discriminate responders to non-responders. Non-responders to nivolumab show high levels of sCD73 activity in serum before treatment, raising the questions whether these patients would benefit from immune checkpoint inhibitors whenever associated with CD73 inhibitors.

## Conclusions

In conclusion we found that the sCD73 enzyme activity in the peripheral blood of melanoma patients may be a potential prognostic factor. The sCD73 enzyme activity, measured before starting treatment, correlates with clinical response to nivolumab. Validation of these results would support the value of sCD73 as predictive biomarker of response to nivolumab therapy, useful to define the subsets of patients who will likely benefit from immunotherapies and possible provide additional treatment target (CD73) in combination with immunotherapeutic agents.

## Abbreviations

sCD73: soluble CD73
CTLA-4: cytotoxic T lymphocyte antigen-4
PD-1: programmed cell death protein-1
ORR: overall response rate
CR: complete response
PR: partial response
SD: stable disease
PD: progressive disease
DCR: disease control rate
CI: confidence interval
HR: hazard ratios
Pi: inorganic phosphate
APCP: adenosine 5’-(a,b-methylene) diphosphate
PTS: patients
WT: wild-type
MUT: mutant
METS: metastasis
LDH: lactate dehydrogenase
IU/L: international units per liter
